# Association between diet quality and overweight or obesity among adults with type 2 diabetes at Debre Markos Comprehensive Specialized Hospital, Northwest Ethiopia

**DOI:** 10.1016/j.obpill.2026.100336

**Published:** 2026-09-21

**Authors:** Samuel Semahgne Abrham, Mengistu Endalamaw, Yonatan Kindie, Desalegn Abebaw, Tilahun Bitew, Temechew Munaw Abebe, Habtamu Belew, Endeshaw Zemen Enatu, Eskeziyaw Agedew, Ayenew Negesse

**Affiliations:** a Department of Human Nutrition, College of Medicine and Health Science, Debre Markos University, Debre Markos, Ethiopia; b Department of Medical Laboratory Sciences, College of Medicine and Health Science, Debre Markos University, Debre Markos, Ethiopia; c Department of Biomedical Science, College of Medicine and Health Science, Debre Markos University, Debre Markos, Ethiopia; d Department of Biomedical Science, College of Medicine and Health Science, Injibara University, Injibara, Ethiopia; e Department of Environmental Health, College of Medicine and Health Science, Debre Markos University, Debre Markos, Ethiopia; f Department of Public Health, College of Medicine and Health Science, Debre Markos University, Debre Markos, Ethiopia

**Keywords:** Diet quality, Overweight, Obesity, Type 2 diabetes mellitus, Physical activity, Glycemic control, Ethiopia

## Abstract

**Background:**

Overweight and obesity are major health challenges among adults with type 2 diabetes mellitus (T2DM), and poor diet quality may contribute to excess body weight. However, evidence regarding the association between overall diet quality and overweight or obesity among adults with T2DM in Ethiopia is limited. This study aimed to assess the association between diet quality and overweight or obesity among adults with T2DM.

**Methods:**

This was an institution-based cross-sectional study conducted among 392 adults with T2DM attending Debre Markos Comprehensive Specialized Hospital, Northwest Ethiopia, from 9 April to May 8, 2026. Participants were selected using systematic random sampling. Data were collected through interviewer-administered questionnaires, medical record review and anthropometric measurements. Diet quality was assessed using the Ethiopia-adapted Diet Quality Questionnaire (DQQ) and Global Dietary Recommendations (GDR) Score. Body Mass Index (BMI) was calculated from measured weight and height and classified according to standard World Health Organization (WHO) categories. Binary logistic regression analyses were conducted to identify factors associated with overweight or obesity. Variables with p ≤ 0.25 in bivariate analysis were included in the multivariate analysis. Adjusted odds ratios (AORs) with 95% confidence intervals (CIs) were used to measure the strength of associations, and statistical significance was declared at p ≤ 0.05.

**Results:**

The prevalence of overweight and obesity was 46.7% (95% CI: 41.5–51.8%) and 7.1% (95% CI: 4.8–10.2%), respectively. In the multivariate analysis, poor diet quality was significantly associated with higher odds of overweight or obesity compared with good diet quality (AOR = 2.46, 95% CI: 1.35–4.44). Being female (AOR = 2.36, 95% CI: 1.34–4.15), monthly income >20,000 Ethiopian birr (AOR = 2.38, 95% CI: 1.26–4.49), physical inactivity (AOR = 2.36, 95% CI: 1.22–4.55) and poor glycemic control (AOR = 8.86, 95% CI: 4.67–16.80) were also significantly associated with higher odds of overweight or obesity.

**Conclusion:**

In this single-center cross-sectional study, more than half of participants met body mass index criteria for overweight or obesity. Poor diet quality, higher reported monthly income, physical inactivity, female sex, and poor glycemic control were associated with higher odds of the combined outcome in the adjusted model. Integrating diet-quality improvement, physical activity promotion, and weight management into routine diabetes care may help address the burden of excess body weight among adults with T2DM.

## Graphical abstract

**Figure:**
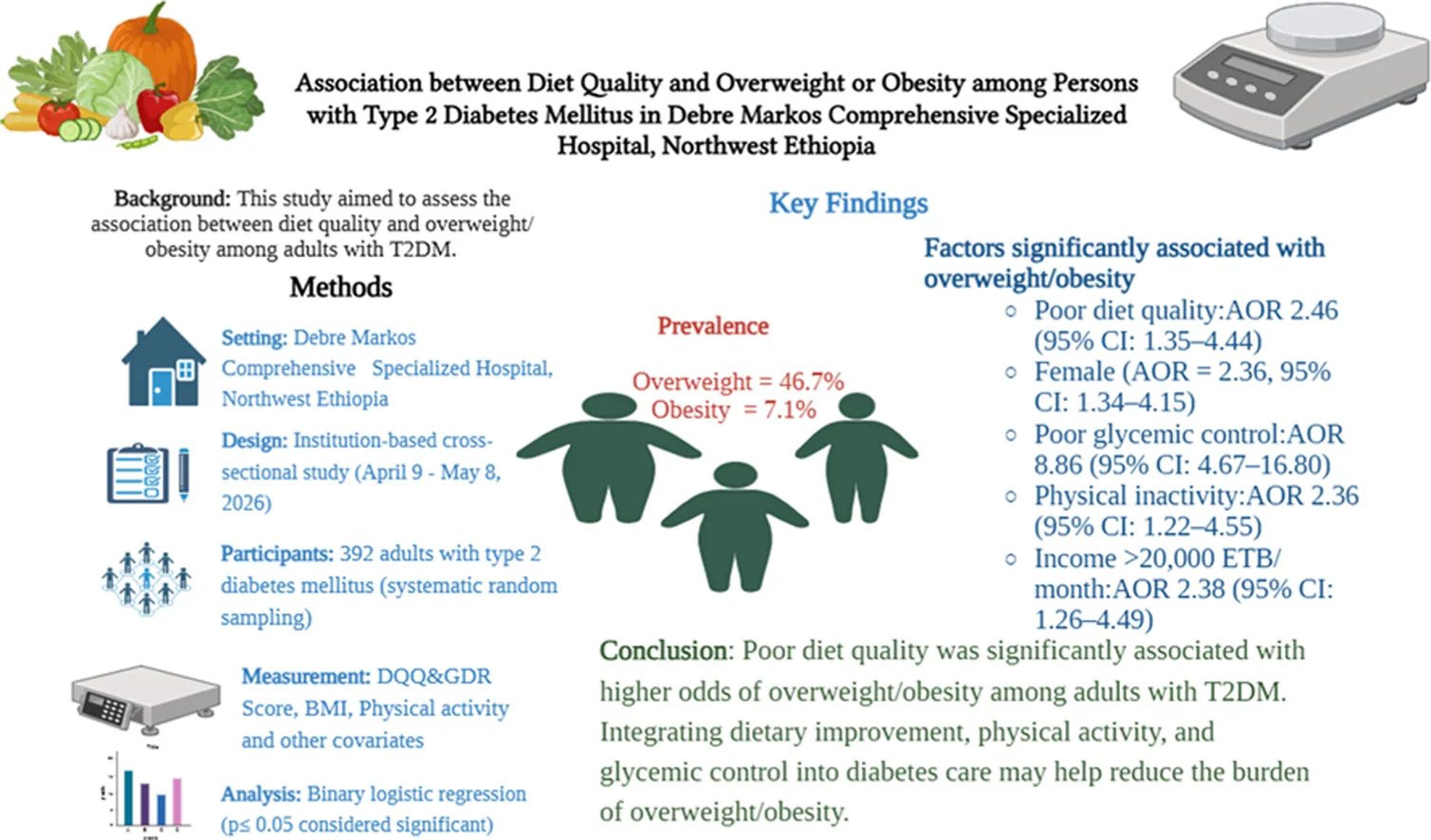

## 1 Introduction

Overweight and obesity are among the most urgent public health challenges worldwide and major contributors to the growing burden of non-communicable diseases (NCDs) [1]. Now, the worsening issue of obesity is classified by the WHO as a chronic, relapsing disease arising from the interplay of genetics, biology, eating behaviors, food access, commercial determinants, and the broader environment [2,3]. Excess adiposity is a well-established risk factor for numerous chronic conditions, including T2DM, cardiovascular disease, hypertension, dyslipidemia, several types of cancer, and premature mortality [4,5]. Changes in dietary patterns, reduced physical activity, urbanization, and other socioeconomic and environmental factors have contributed to rising levels of overweight and obesity among adults with diabetes [6,7]. Given these adverse health consequences, international diabetes management guidelines recommend weight management as a cornerstone of T2DM care, alongside optimal glycemic control and other lifestyle interventions [3].

Diet quality has emerged as a key modifiable determinant of obesity and metabolic health [8]. It reflects the overall healthfulness of dietary intake and adherence to dietary recommendations rather than focusing on individual foods or nutrients [9]. Higher diet quality, characterized by greater consumption of fruits, vegetables, legumes, whole grains and other nutrient-dense foods with lower consumption of unhealthy foods, has been associated with healthier body weight, improved insulin sensitivity, better glycemic control, and reduced cardiometabolic risk [10,11]. Conversely, poor diet quality contributes to excess energy intake, weight gain, and the development and progression of obesity and T2DM [12,13].

The coexistence of excess body weight and T2DM has become increasingly common, particularly in low- and middle-income countries, reflecting the broader nutritional and epidemiological transition across many populations [6,14]. Ethiopia is experiencing a growing burden of overweight, obesity, and diet-related NCDs alongside persistent undernutrition [15,16]. Evidence from a study indicates that the prevalence of overweight and obesity has increased over recent decades, particularly in urban populations, reflecting changes in dietary patterns, physical inactivity, and socioeconomic conditions [17].

People with T2DM are particularly vulnerable to overweight and obesity as a result of multifactorial interactions involving insulin resistance, lifestyle-related factors, pharmacological effects and other metabolic abnormalities [3]. Previous evidence from Ethiopia has identified a substantial association between overweight and obesity and diabetes mellitus, emphasizing excess adiposity as an important factor in the diabetes burden [18]. Despite this evidence, little is known about whether overall diet quality is associated with overweight and obesity among Ethiopian adults already living with T2DM. Most previous studies have focused on obesity magnitude, glycemic control, or individual dietary components rather than evaluating the relationship between comprehensive diet quality and excess body weight in this high-risk population.

Assessing the association between diet quality and overweight or obesity among adults with T2DM is important because it may identify modifiable dietary factors associated with excess body weight, providing opportunities to strengthen nutrition counseling and support more effective lifestyle-based obesity prevention and weight management. Such evidence can inform nutrition counseling, lifestyle interventions, and diabetes care programs aimed at improving both metabolic health and obesity-related outcomes. Therefore, this study aimed to assess the association between diet quality and overweight or obesity among adults with type 2 diabetes mellitus attending Debre Markos Comprehensive Specialized Hospital in Northwest Ethiopia.

## 2 Methods and materials

### 2.1 Study design and period

An institution-based cross-sectional study was conducted among adults with T2DM attending Debre Markos Comprehensive Specialized Hospital. The hospital provides a range of preventive, curative, and rehabilitative services. The study was conducted from 9 April to May 8, 2026.

### 2.2 Eligibility criteria

Adults aged ≥18 years with type 2 diabetes mellitus were eligible for inclusion in the study. Participants who were seriously ill, had a chronic disease associated with edema, were pregnant, or had a psychiatric disorder were excluded from the study.

### 2.3 Sample size and sampling technique

The required sample size was calculated using a single-population proportion formula, considering an expected prevalence of 36.2% from a previous study [19], a 95% CI, and a 5% margin of error. The initial sample size was calculated to be 356; by adding a 10% non-response rate, the final sample was 392 participants. Systematic random sampling was used to select eligible participants attending treatment follow-up in the hospital. Eligibility was verified before selection according to the predefined inclusion and exclusion criteria. Each participant was selected only once to avoid duplication from repeat clinic visits. When a selected participant was ineligible or declined participation, the next eligible participant was selected as a replacement, and the systematic sampling procedure was continued thereafter. A total of 392 participants with T2DM were selected from 1980 T2DM patients receiving treatment follow-up. A random starting point was selected from the first five eligible participants, after which every fifth eligible participant was selected.

## 3 Data collection method

### 3.1 Data collection tool and procedures

Data were collected using an interview-administered questionnaire, reviewing medical charts, and taking anthropometric measurements by trained data collectors. The questionnaire was programmed and administered using KoboToolbox. All data collectors received training on the study's objective, proper interview techniques, data extraction from charts, standardization of measurement techniques, and ethical considerations. Trained supervisors conducted regular supervision to ensure the accuracy and completeness of data. In order to check the clarity of the tool and variables, pre-tests were done. The tool included sections that mainly consisted of closed- and open-ended questions and was delivered to eligible participants. The questionnaire consisted of socio-demographic characteristics, diet quality assessment, household food insecurity, medication adherence, physical activity, behavioral and diabetes-related factors and nutrition knowledge.

Food insecurity was assessed using the Household Food Insecurity Access Scale (HFIAS), developed by the Food and Nutrition Technical Assistance (FANTA) project, to measure household food insecurity. Medication adherence was assessed by using the 8-item Morisky Medication Adherence Scale (MMAS-8). A nutrition knowledge questionnaire adapted from a study in Ghana on nutrition knowledge for patients with diabetes was used to assess the knowledge of participants. Physical activity was assessed using the Global Physical Activity Questionnaire (GPAQ). Medical information charts were reviewed to record the participants' blood pressure and the current and last two months' fasting blood sugar levels.

### 3.2 Anthropometric measurements

Anthropometric measurements were obtained following standardized procedures using calibrated equipment. Height was measured to the nearest 0.1 cm using a standard stadiometer, with patients standing upright without shoes. Participants were instructed to maintain a straight posture, face forward, and place both heels together on the floor. The head was positioned so that the Frankfurt horizontal plane was maintained before the measurement was taken. Body weight was measured to the nearest 0.1 kg using a calibrated weighing scale. Participants were instructed to remove heavy outer clothing, scarves, and items from their pockets before measurement and to stand with both feet close together at the center of the scale. Body mass index (BMI) was subsequently computed by dividing body weight in kilograms by the square of height in meters (kg/m^2^) [20].

### 3.3 Assessment of diet quality

Diet quality was assessed using the Diet Quality Questionnaire (DQQ) of Ethiopia developed by the Global Diet Quality Project (GDQP) [21]. The DQQ uses a 24-h dietary recall to assess the consumption of major food groups and food categories. An indicator of diet quality Global Dietary Recommendations Score (GDRS), which is composed of two sub-metrics (the GDR-Healthy score and the GDR-Limit score, recently renamed NCD-Protect and NCD-Risk), based on the consumption of 17 food groups during the past day and night, and is calculated as follows: GDR-Healthy - GDR-Limit + 9 = GDR score. A GDR score ≥10 indicates good diet quality and is associated with meeting at least 6 out of the 11 global dietary recommendations [22]. These good and poor diet-quality categories were operational definitions based on the established GDRS threshold and were not intended as clinical classifications of individual dietary intake.

### 3.4 Operational definitions

**Weight status:** Was classified based on BMI category participants as underweight if BMI was under 18.5 kg/m^2^, normal weight if BMI was between 18.5 and 24.9 kg/m^2,^ and overweight if BMI was from 25 kg/m^2^ to 29.9 kg/m^2^ and obese if BMI greater or equal to 30 kg/m^2^ [23].

**Blood pressure**: Medical information charts were reviewed to record participants’ blood pressure. Participants were classified as having normal blood pressure if their systolic and diastolic blood pressure measurements were <120/80 mm Hg, elevated blood pressure if there were 120–129/< 80 mm Hg, stage 1 hypertension if they were 130–139 mm Hg (systolic) or 80–89 mm Hg (diastolic) and stage 2 hypertension if they were ≥140 mm Hg (systolic) or ≥ 90 mm Hg (diastolic) [24].

**Glycemic control:** Glycemic control was assessed using the average of three consecutive readings, including the current and the previous two readings. Fasting blood glucose (FBG) of 80–130 mg/dl was classified as good glycemic control, and FBG >130 mg/dl as poor glycemic control [25]. FBG <80 mg/dl was also considered poor glycemic control.

**Food insecurity:** Households were classified as food secure, mildly food insecure, moderately food insecure, and severely food insecure according to the guidelines, and for analysis, it was classified as food secure (HFIAS = 0) and food insecure (mildly food insecure, moderately food insecure, and severely food insecure) [26].

**Nutrition knowledge:** Any respondent who answered 12 or more questions from a total of 16 questions was considered to have adequate knowledge, while those scoring below 12 were considered to have inadequate knowledge [27].

**Adequate physical activity:** Non-adherent to physical activity recommendations if participants scored less than 600 Metabolic Equivalent of Task (MET)-minutes per week, based on the Global Physical Activity Questionnaire (GPAQ) for physical activity integrated scoring method and adherent to physical activity recommendations if participants scored 600 MET-minutes per week or more, based on the GPAQ integrated scoring method [28].

**Current alcohol use:** Self-reported consumption of alcoholic drinks commonly in the past 30 days.

**Medication adherence:** Participants were categorized as having high adherence (score = 0), medium adherence (score = 1–2), or low adherence (score = 3–8). For the regression analysis, participants with high adherence were classified as adherent, whereas those with medium or low adherence were classified as non-adherent [29].

**Presence of comorbidities:** Participants who had at least one other chronic non-communicable disease/s that was previously diagnosed [30].

**Central obesity:** A participant was classified as having central obesity if their waist circumference ≥94 cm for males and ≥80 cm for females [31].

### 3.5 Statistical analysis

Data were extracted from Kobo Toolbox, exported to Excel, and then imported into IBM SPSS Statistics Version 27 for data cleaning and analysis. Descriptive statistics, including frequencies, percentages, summary measures (means and standard deviations) and crosstabs were applied to summarize data. Internally consistent nutrition knowledge questionnaires were used to assess participants' nutrition knowledge. A Cronbach's alpha of 0.7 or higher indicates that the questionnaire items reliably measure nutrition knowledge. Multicollinearity among independent variables was assessed using the Variance Inflation Factor (VIF). The Hosmer-Lemeshow goodness-of-fit test was conducted to check each model's fitness. The final multivariable logistic regression model was also checked for discrimination between participants with and without overweight or obesity in an area under the receiver operating characteristic curve (AUC) [32]. Variables with a p-value ≤0.25 in bivariable logistic regression were considered candidates for the multivariable model. All eligible variables were entered simultaneously into the multivariable logistic regression model to identify factors independently associated with overweight or obesity. Complete-case analysis was used, and participants with missing values for variables included in the model were excluded. Model assumptions, including linearity of continuous predictors and potential interactions, were assessed before interpreting the final model. Missing data were assessed for all study variables before analysis. Participants with missing values for variables included in the multivariable logistic regression model were excluded from the corresponding analysis using complete-case analysis. The number of observations included in the final multivariable model was reported in Table 3. Statistical significance was defined at a p-value ≤0.05 and the odds ratio, along with the 95% CI estimate, was used to measure the strength of the association.

### 3.6 Ethical approval and participant consent

Ethical clearance was obtained from the Debre Markos University College of Medicine and Health Sciences Research Ethics Committee (RCTTD/666/01/18). Participants were clearly informed about the study and provided written informed consent before participation. The data collector explained the purpose of the study in a language they could understand, as well as their right to participate or refuse without any impact on the services they received. Thus, only those who gave consent to participate were interviewed and measured. In addition, privacy was maintained during data collection, and the confidentiality of all information provided was strictly maintained. The study procedures were conducted in accordance with the Declaration of Helsinki.

## 4 Results

### 4.1 Socio-demographic and socio-economic characteristics

A total of 392 adults with T2DM participated in the study. The mean age of the participants was 53.4 ± 11.3 years. More than half of the participants (55.1%) were male. Regarding residence, 63.5% of the participants were from rural areas and the majority of participants (79.3%) were Orthodox Christians. Regarding occupation, 42.3% of the participants were farmers, followed by government or private employees (27.6%) and merchants (14.5%). In terms of monthly income, 40.1% of participants reported an income of less than 10,000 Ethiopian birr, while 39.8% reported an income greater than 20,000 Ethiopian birr. Approximately half of the participants (49.0%) were from food-secure households and 37.0% of participants had graduated from college or university (Table 1).

**Table 1:** Socio-demographic characteristics of adults with type 2 diabetes mellitus attending Debre Markos Comprehensive Specialized Hospital, Northwest Ethiopia, 2026 (n = 392).

Variables	Category	Frequency	Percentage
Age	Less than 44	105	26.8
	45–65	226	57.6
	Above 65	61	15.6
Sex	Male	216	55.1
	Female	176	44.9
Residences	Rural	249	63.5
	Urban	143	36.5
Religion	Orthodox	311	79.3
	Muslim	37	9.5
	Protestant	44	11.2
Occupation	Merchant	57	14.5
	Farmer	166	42.3
	Government or private employee	108	27.6
	Housewife	31	7.9
	Daily Laborer	30	7.7
Income category	Less than 10,000	157	40.0
	10,000–20,000	79	20.2
	Greater than 20,000	156	39.8
Household food insecurity	Secure	192	49.0
	Insecure	200	51.0
Education	Cannot read and write	56	14.3
	Can read and write	32	8.2
	Primary	119	30.4
	Secondary	40	10.2
	College or university graduate	145	36.9

### 4.2 Behavioral and clinical characteristics

Among the study participants, 235 (59.9%) had good diet quality and 123 (31.4%) participants had good nutrition knowledge. Regarding physical activity, 114 (29.1%) participants were active, while 278 (70.9%) were inactive. Among the study participants, 182 (46.4%) participants had controlled glycemic levels and 198 (50.5%) had normal blood pressure, 73 (18.6%) had elevated blood pressure, 90 (23.0%) had stage 1 hypertension, and 31 (7.9%) had stage 2 hypertension. With respect to medication use and adherence, 6 (1.5%) participants were not taking their prescribed medication, and 281 (71.7%) were adherent. Regarding comorbidities, 297 (75.8%) participants had no comorbidities, whereas 95 (24.2%) had at least one comorbid condition. Furthermore, 217 (55.4%) participants had no central obesity, while 175 (44.6%) had central obesity (Table 2).

**Table 2:** Behavioral and clinical characteristics of adults with type 2 diabetes mellitus attending Debre Markos Comprehensive Specialized Hospital, Northwest Ethiopia, 2026 (n = 392).

Variables	Category		Frequency	Percentage
Diet quality	Good		235	59.9
	Poor		157	40.1
Nutrition knowledge status	Good		123	31.4
	Poor		269	68.6
Physical activity	Active		114	29.1
	Inactive		278	70.9
Glycemic control	Controlled		182	46.4
	Not controlled		210	53.6
Blood pressure	Normal		198	50.5
	Stage 1 HTN		90	23.0
	Stage 2 HTN		31	7.9
	Elevated BP		73	18.6
Medication use	No		6	1.5
	Yes	Adherent	281	71.7
		Non-adherent	105	26.8
Comorbidity	No		297	75.8
	Yes		95	24.2
Central obesity	Absent		217	55.4
	Present		175	44.6

### 4.3 Prevalence of overweight and obesity

The prevalence of overweight and obesity was 46.7% (95% CI: 41.5–51.8%) and 7.1% (95% CI: 4.8–10.2%), respectively. The overall prevalence of both overweight and obesity was 53.8% (95% CI: 48.8–58.8%).

### 4.4 Factors associated with overweight and obesity

In the bivariate logistic regression analysis, sex, residence, monthly income, household food insecurity, diet quality, comorbidity, physical activity, and glycemic control were associated with overweight or obesity and were therefore included in the multivariate analysis. In the multivariate logistic regression analysis, sex, monthly income, diet quality, physical activity, and glycemic control remained significantly associated with overweight or obesity. Central obesity, assessed using waist circumference, was not included as a candidate predictor in the regression analysis because it is closely related to the BMI-based overweight or obesity outcome and may result in conceptual overlap between the predictor and outcome. Multicollinearity among the independent variables was assessed using the variance inflation factor (VIF). No evidence of problematic multicollinearity was observed, as all VIF values were less than 2. The Hosmer–Lemeshow goodness-of-fit test showed that the model demonstrated good fit (p = 0.828). The final multivariable logistic regression model demonstrated AUC of 0.869, indicating substantial discrimination between participants with and without overweight or obesity (Fig. 1).

**Fig. 1:**
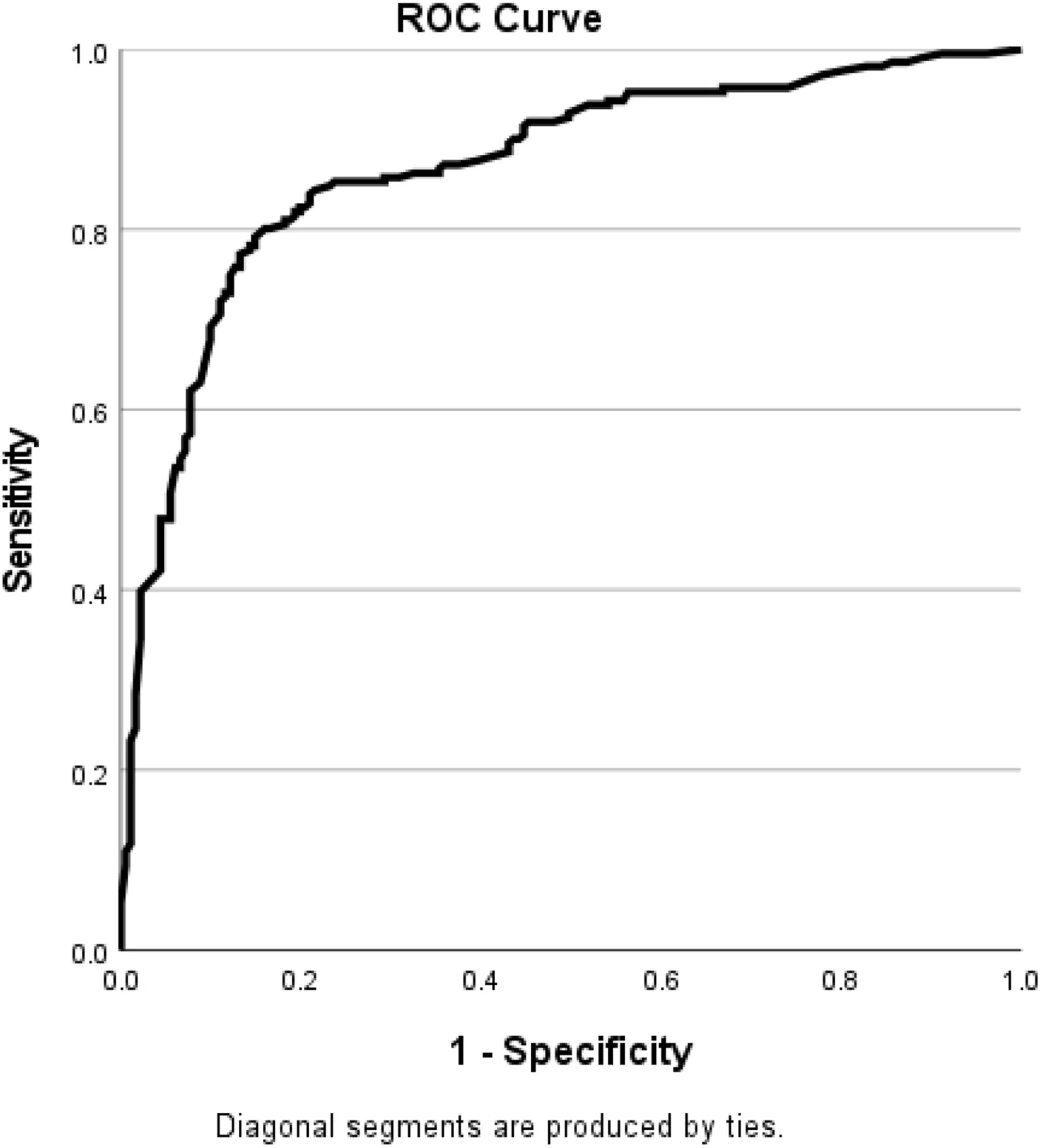
Receiver operating characteristic (ROC) curve for the final multivariable logistic regression model predicting overweight or obesity.

The sensitivity analysis showed that the association between poor glycemic control and overweight or obesity remained consistent with the primary analysis. The effect estimate was very close in magnitude and direction to the primary model estimate (AOR = 8.86, 95% CI: 4.67–16.80, p < 0.001), indicating that the observed association was robust to the sensitivity analysis.

Patients with T2DM with poor diet quality had approximately 2.5 times higher odds of having overweight or obesity compared with those with good diet quality (AOR = 2.46, 95% CI: 1.35–4.44). Female patients with T2DM had higher odds of having overweight or obesity than their counter parts (AOR = 2.36, 95% CI: 1.34–4.15). Patients with T2DM with a monthly income greater than 20,000 ETB had higher odds of having overweight or obesity compared with those earning less than 10,000 ETB (AOR = 2.38, 95% CI: 1.26–4.49). Physical inactivity was also significantly associated with overweight or obesity. Physically inactive patients with T2DM had more than twice the odds of having overweight or obesity compared with physically active patients with T2DM (AOR = 2.36, 95% CI: 1.22–4.55). Furthermore, patients with T2DM with poor glycemic control had nearly 9 times higher odds of having overweight or obesity compared with those with good glycemic control (AOR = 8.86, 95% CI: 4.67–16.80) (Table 3).

**Table 3:** Bivariate and multivariate analysis of factors associated with overweight or obesity among adults with type 2 diabetes mellitus attending Debre Markos Comprehensive Specialized Hospital, Northwest Ethiopia, 2026 (n = 392).

Variables	Categories	Overweight or Obesity		COR (95% CI)	AOR (95% CI)	p-value
		Yes	No			
Diet quality	Poor	123	34	6.04 (3.81–9.60)	**2.46(1.35**–4.44)*	**0.003**
	Good	88	147	1	1	
Sex	Female	101	75	1.30 (0.67–1.94)	**2.36(1.34**–4.15)*	**0.003**
	Male	110	106	1.00	1.00	
Residences	Rural	147	102	1.00	1.00	
	Urban	64	79	1.78 (1.17–2.70)	1.00 (0.56–1.78)	0.988
Monthly income in ETB	<10,000	62	95	1.00	1.00	
	10,000–20000	43	36	1.83 (1.06–3.16)	1.54 (0.75–3.17)	0.245
	>20,000	106	50	3.25 (2.04–5.17)	**2.38(1.26**–4.49)*	**0.008**
Household food security	Secure	124	68	2.37 (1.58–3.56)	1.07 (0.60–1.90)	0.814
	Insecure	87	113	1.00	1.00	
Comorbidity	Yes	63	32	1.98 (1.22–3.21)	1.62 (0.84–3.11)	0.151
	No	148	149	1.00	1.00	
Physical activity	Inactive	186	92	7.20 (4.33–11.98)	**2.36(1.22**–4.55)*	**0.011**
	Active	25	89	1.00	1.00	
Glycemic control	Poor	173	37	17.72 (10.71–29.32)	**8.86(4.67**–16.80)*	**<0.001**
	Good	38	144	1.00	1.00	

**Bold** and * indicate factors significantly associated at p < 0.05.

## 5 Discussion

The objective of this study was to determine the association between diet quality and overweight or obesity among adults with T2DM attending Debre Markos Comprehensive Specialized Hospital in Northwest Ethiopia. The prevalence of overweight and obesity was 46.7% (95% CI: 41.5–51.8%) and 7.1% (95% CI: 4.8–10.2%), respectively and the overall prevalence of both overweight and obesity was 53.8% (95% CI: 48.8–58.8%). Importantly, participants with poor diet quality had significantly higher odds of having overweight or obesity than those with good diet quality. In addition, higher monthly income, physical inactivity, and poor glycemic control were significantly associated with overweight or obesity.

The prevalence of overweight and obesity observed in this study indicates a substantial burden of excess body weight among adults with T2DM. Although the present study also showed a prevalence of overweight or obesity among patients with T2DM consistent with the study in Sidama and Wolayta, which reported 55.1% and 49.9%, this study revealed a higher prevalence than studies in Hosanna, Mekelle and Jimma [19,33,34]. The higher prevalence observed in the present study may partly reflect differences in study setting, population characteristics, dietary practices, socioeconomic conditions, physical activity patterns, and the timing of the studies. The present study was conducted in 2026, whereas the previous studies were conducted earlier, and the increasing nutrition transition and changes in food environments may also contribute to differences in the burden of excess body weight. Nevertheless, overweight or obesity among patients with T2DM in the present study was lower than reported in previous studies from Saudi Arabia (81.6%), Tanzania (85.0%), Uganda (63.0%) and Nigeria (62.9%) [[35], [36], [37], [38]]. The lower prevalence compared with these international studies may reflect differences in population characteristics, socioeconomic conditions, dietary patterns, physical activity, healthcare systems, and the distribution of obesity-related risk factors across settings.

The most important finding of this study was the significant association between diet quality and overweight or obesity. Participants with poor diet quality had increased odds of having overweight or obesity compared with participants with good diet quality. This finding is consistent with a study in Iran among patients with T2DM, which found that men with higher BMI had significantly lower adapted Healthy Eating Index scores, indicating poorer overall diet quality, while higher diet quality was associated with lower BMI [39]. This study is also consistent with a study in China, where better diet quality, as characterized by higher Alternate Healthy Eating Index (AHEI) scores, was significantly associated with lower odds of obesity after adjustment for other factors [40]. The consistency of the findings across substantially different populations suggests that overall dietary quality may be relevant to weight management among people living with T2DM. Nevertheless, the present study cannot establish whether poor diet quality preceded weight gain or whether participants with overweight or obesity changed their diets in response to their diabetes or weight status. Reverse association is therefore possible.

In the present study, female patients with T2DM had higher odds of being overweight or obesity. This is consistent with a study conducted among patients with T2DM in Morocco, which reported that female sex was significantly associated with general obesity [41]. Similarly, a hospital-based study among patients with T2DM in Eastern Ethiopia reported a higher mean BMI among women than men, supporting the greater burden of excess body weight among female patients with T2DM [42]. These findings may reflect sex-related differences in body-fat distribution, physical activity, dietary patterns, reproductive and hormonal factors, and sociocultural roles that may influence opportunities for physical activity.

An increase (>20,000 ETB) in reported monthly income was associated with higher odds of overweight or obesity among patients with type 2 diabetes in this study, consistent with studies in Uganda and Southwest Ethiopia [19,37]. The positive association may reflect greater purchasing power among higher-income individuals, which may provide greater access to energy-dense and processed foods and may be accompanied by more sedentary lifestyles. In this study, patients with type 2 diabetes who were physically inactive also had higher odds of overweight or obesity, consistent with prior evidence showing an inverse association between physical activity and body weight among individuals with type 2 diabetes [19,33,34,43]. The association may reflect lower energy expenditure among physically inactive individuals, which can contribute to positive energy balance, fat accumulation, and subsequent overweight or obesity.

In this study, participants with poor glycemic control had nearly nine times the odds of having overweight or obesity compared with those with good glycemic control. This strong association aligns with a study in patients with T2DM that found weight gain was associated with HbA1c across 12 countries in Europe, Canada and Japan. The possible explanation is that poor glycemic control may be accompanied by behavioral and treatment-related factors that influence body weight. Some glucose-lowering medications can also affect body weight. The observed association between glycemic control and overweight or obesity may be explained by the close bidirectional relationship between body weight and glucose metabolism. A reverse association was also observed in several studies in India, Europe, Australia, and Ethiopia, including in Dire Dawa, Jimma, Adama, and the Amhara region, where poorer glycemic control was observed among patients with overweight or obesity with T2DM [[44], [45], [46], [47], [48], [49]]. This may be explained as individuals with overweight and obesity contribute to insulin resistance by releasing pro-inflammatory cytokines and free fatty acids, which interfere with insulin signaling pathways [[50], [51], [52]].

However, because the present study was cross-sectional, the temporal direction of the observed association cannot be established, and the relationship may be bidirectional. Therefore, our finding should be interpreted as an association rather than evidence that poor glycemic control causes overweight or obesity.

### 5.1 Limitation of the study

Although diet quality was assessed using the Ethiopia-adapted Diet Quality Questionnaire, which provides a structured assessment of overall diet quality in the local context, the following limitations of the study should be acknowledged. First, dietary assessment was based on a 24-h dietary recall, which may not fully represent participants’ usual dietary intake and may be affected by recall and reporting bias. Second, the study was conducted in a single hospital, which may limit the generalizability of the findings to patients with T2DM in other hospitals or regions of Ethiopia. Third, physical activity, alcohol use and medication adherence variables were self-reported and may be affected by recall or social desirability bias. Fourth, important residual variables may not have been included in the multivariable model. Fifth, glycemic control was assessed using FBG, which may be influenced by short-term factors and provide a less comprehensive measure of long-term glycemic control than HbA1c, and some misclassification of glycemic control may have occurred. Therefore, direct comparison with studies using HbA1c-based definitions of glycemic control should be made cautiously, as these measures reflect different aspects of glycemic status. Six, overweight or obesity was classified using body mass index alone, which does not distinguish between fat mass and lean mass and may therefore have resulted in some outcome misclassification. Seven, treatment-related confounding may also have influenced the observed associations, particularly because specific glucose-lowering therapies were not accounted for in the analysis. Finally, although the GDRS has demonstrated applicability among adults and has been evaluated as an indicator of adherence to global dietary recommendations, the DQQ was primarily developed for population-level diet-quality monitoring rather than comprehensive assessment of individual dietary intake. Therefore, the good and poor diet-quality categories used in this study should be interpreted as operational indicators of adherence to global dietary recommendations rather than clinical classifications of individual diet quality. In addition, specific validation of the GDRS ≥10 classification among adults with type 2 diabetes mellitus in Ethiopia has not been established, which may limit the interpretation and generalizability of the findings. Further studies using quantitative dietary assessment methods are warranted to evaluate the performance of the GDRS and its threshold in adults with type 2 diabetes mellitus in Ethiopia.

## 6 Conclusion

The study demonstrated a substantial burden of excess body weight among patients with T2DM attending Debre Markos Comprehensive Specialized Hospital, with more than half of participants classified as overweight or obese. Poor diet quality was significantly associated with higher odds of overweight or obesity, which may highlight the potential importance of overall dietary quality in weight management. Female sex, higher monthly income, physical inactivity and poor glycemic control were also significantly associated with overweight or obesity. However, the cross-sectional design precludes establishing a temporal or causal relationship. Longitudinal and intervention studies are needed to clarify the temporal relationship between diet quality and overweight or obesity.

## Clinical takeaway messages

• Overweight or obesity affected greater than half of adults with T2DM, highlighting the need for routine weight assessment and management in diabetes care.
• Poor diet quality was associated with higher odds of overweight or obesity, supporting integrated dietary counseling.
• Physical inactivity and poor glycemic control were strongly associated with overweight or obesity, emphasizing the importance of integrated physical-activity promotion and glycemic management in routine T2DM care.

## Data availability

The data used are available from the corresponding author upon request.

## Clinical trial number

Not applicable.

## Author contributions

SSA: Conceptualization, methodology, investigation, data curation, formal analysis, visualization, writing—original draft and writing—review and editing. ME: Methodology, investigation, data collection, supervision and writing—review and editing. YK: Investigation, data collection, data curation and writing—review and editing. DA: Investigation, data collection and writing—review and editing. TB: Methodology, investigation and writing—review and editing. TMA: Investigation, Methodology and writing—review and editing. HB: Conceptualization, methodology, supervision and writing—review and editing. EZE: Methodology, data collection, supervision and writing—review and editing. EA: Investigation, data collection and writing—review and editing. AN: Conceptualization, methodology, supervision and writing—review and editing.

All authors contributed to the interpretation of the findings, critically reviewed the manuscript, approved the final version and agreed to be accountable for all aspects of the work.

## Declaration of AI use

The authors used ChatGPT for checking grammatical errors or improving clarity during preparation of the original manuscript. All ChatGPT-assisted content was reviewed, edited, and verified by the human authors, who take full responsibility for the integrity of the work.

## Funding

No funding.

## Conflict of interest

No potential conflicts of interest reported by authors.

## Abbreviations

DQQ: Diet Quality Questionnaire
GDQP: Global Diet Quality Project
GDR: Global Dietary Recommendations
GPAQ: Global Physical Activity Questionnaire
MMAS-8: 8-item Morisky Medication Adherence Scale
T2DM: Type 2 Diabetes Mellitus
WHO: World Health Organization

## References

[1] Organization WH (2026). The WHO acceleration plan to stop obesity: a joint WHO/UNICEF operational model for designing and implementing the response.

[2] Organization WH (2025). https://www.who.int/en/news-room/fact-sheets/detail/obesity-and-overweight.

[3] (2026). 8. Obesity and weight management for the prevention and treatment of diabetes: standards of care in diabetes–2026. Diabetes Care.

[4] Powell-Wiley T.M., Poirier P., Burke L.E., Després J.-P., Gordon-Larsen P., Lavie C.J. (2021). Obesity and cardiovascular disease: a scientific statement from the American heart association. Circulation.

[5] Suárez R., Sarno G., Andrade C., Gomez-Correa D., Jima D., Matos A. (2026). Global trends in obesity-related cancer: risk factors, prevention, and policy interventions. Food Agric Immunol.

[6] Ekpor E., Akyirem S., Adade Duodu P. (2023). Prevalence and associated factors of overweight and obesity among persons with type 2 diabetes in Africa: a systematic review and meta-analysis. Ann Med.

[7] Popkin B.M., Ng S.W. (2022). The nutrition transition to a stage of high obesity and noncommunicable disease prevalence dominated by ultra-processed foods is not inevitable. Obes Rev.

[8] Angelico F., Baratta F., Coronati M., Ferro D., Del Ben M. (2023). Diet and metabolic syndrome: a narrative review. Intern Emerg Med.

[9] Miller V., Webb P., Micha R., Mozaffarian D. (2020). Defining diet quality: a synthesis of dietary quality metrics and their validity for the double burden of malnutrition. Lancet Planet Health.

[10] Reger C., Leitzmann M.F., Rohrmann S., Kühn T., Sedlmeier A.M., Jochem C. (2024). Sustainable diets and risk of overweight and obesity: a systematic review and meta-analysis. Obes Rev.

[11] Taylor R.M., Haslam R.L., Herbert J., Whatnall M.C., Trijsburg L., de Vries J.H.M. (2024). Diet quality and cardiovascular outcomes: a systematic review and meta-analysis of cohort studies. Nutr Diet.

[12] Mokhtari Z., Abdollahzad H., Rezaeian S., Izadi N., Darbandi M., Najafi F. (2024). International dietary quality index and its association with diabetes in RaNCD cohort study. Sci Rep.

[13] Fanelli S.M., Jonnalagadda S.S., Pisegna J.L., Kelly O.J., Krok-Schoen J.L., Taylor C.A. (2020). Poorer diet quality observed among US adults with a greater number of clinical chronic disease risk factors. J Prim Care Community Health.

[14] Abrham S.S., Jemal M., Ashenef B., Tiruneh B., Kase M., Belew H. (2026). Prevalence of overweight and obesity and associated factors among persons with type 2 diabetes mellitus in Ethiopia: a systematic review and meta-analysis. Obes Pillars.

[15] Kassie A.M., Abate B.B., Kassaw M.W. (2020). Prevalence of overweight/obesity among the adult population in Ethiopia: a systematic review and meta-analysis. BMJ Open.

[16] Yisak H., Zemene M.A., Arage G., Demelash A.T., Anley D.T., Ewunetei A. (2023). Undernutrition and associated factors among older adults in Ethiopia: systematic review and meta-analysis. BMJ Open.

[17] Azanaw M.M., Zewde E.A., Gebremariam A.D., Dagnaw F.T., Asnakew D.T., Chanie E.S. (2022). Spatiotemporal distribution and determinants of overweight or obesity among urban women in Ethiopia: a multivariate decomposition analysis. BMC Womens Health.

[18] Muche Ewunie T., Sisay D., Kabthymer R.H. (2022). Diabetes mellitus and its association with central obesity, and overweight/obesity among adults in Ethiopia. A systematic review and meta-analysis. PLoS One.

[19] Abdissa D., Dukessa A., Babusha A. (2021). Prevalence and associated factors of overweight/obesity among type2 diabetic outpatients in southwest Ethiopia. Heliyon.

[20] Moltrer M., Pala L., Cosentino C., Mannucci E., Rotella C., Cresci B. (2022). Body mass index (BMI), waist circumference (WC), waist-to-height ratio (WHtR) e waist body mass index (wBMI): which is better?. Endocrine.

[21] Project GDQ Diet quality questionnaire (DQQ) for Ethiopia. https://www.dietquality.org/countries/eth.

[22] Frongillo E., Coates J., Verger E.O., Savy M., Martin-Prével Y., Neufeld L. (2023).

[23] Weir C.B., Jan A. (2019).

[24] Whelton P.K., Carey R.M., Aronow W.S., Casey D.E., Collins K.J., Dennison Himmelfarb C. (2018). 2017 ACC/AHA/AAPA/ABC/ACPM/AGS/APhA/ASH/ASPC/NMA/PCNA guideline for the prevention, detection, evaluation, and management of high blood pressure in adults: executive summary: a report of the American college of cardiology/American heart association task force on clinical practice guidelines. Circulation.

[25] MoH F. (2016).

[26] Coates J., Swindale A., Bilinsky P. (2007).

[27] Dawuda A.S., Abdul-Aziz A. (2023). Nutritional knowledge and dietary habits among diabetic patients at the Bono regional hospital, Sunyani, Ghana. J Adv Med Pharmaceut Sci.

[28] Organization W.H. (2021).

[29] Tandon S., Chew M., Eklu-Gadegbeku C.K., Shermock K.M., Morisky D.E. (2015). Validation and psychometric properties of the 8-item morisky medication adherence scale (MMAS-8) in type 2 diabetes patients in Sub-Saharan Africa. Diabetes Res Clin Pract.

[30] Ejeta A., Abdosh T., Hawulte B., Lamessa A., Belete Fite M., Fekadu G. (2021). Diabetes concordant comorbidities and associated factors among adult diabetic out-patients at Hiwot Fana specialized university hospital, harar, eastern Ethiopia: a cross-sectional study. Diabetes Metab Syndr Obes.

[31] Consultation W. (2008).

[32] Alba A.C., Agoritsas T., Walsh M., Hanna S., Iorio A., Devereaux P. (2017). Discrimination and calibration of clinical prediction models: users' guides to the medical literature. JAMA.

[33] Lambebo A., Egata G., Mangistie B., Irenso A.A. (2018). Overweight and obesity among type 2 diabetic patients at nigist elleni memorial hospital, hosanna town, southern Ethiopia. East Afr J Health Biomed Sci.

[34] Kiros K.G., Abyu G.Y., Belay D.S., Goyteom M.H., Welegebriel T.K. (2019). Magnitude of overweight and associated factors among type 2 diabetes mellitus patients at Mekelle public hospitals, Tigray, Ethiopia: a cross-sectional study. BMC Res Notes.

[35] Jarad Fas Al, Narapureddy B.R., Derkaoui H.R., Aldayal A.S.A., Alotaibi M.M.H., Aladhyani F.H.A. (2025). Prevalence and risk factors of obesity among type 2 diabetic participants in Abha, Saudi Arabia: a cross-sectional study. Healthcare (Basel).

[36] Damian D.J., Kimaro K., Mselle G., Kaaya R., Lyaruu I. (2017). Prevalence of overweight and obesity among type 2 diabetic patients attending diabetes clinics in northern Tanzania. BMC Res Notes.

[37] Tino S., Mayanja B.N., Mubiru M.C., Eling E., Ddumba E., Kaleebu P. (2020). Prevalence and factors associated with overweight and obesity among patients with type 2 diabetes mellitus in Uganda—A descriptive retrospective study. BMJ Open.

[38] Gezawa I.D., Uloko A.E., Gwaram B.A., Ibrahim D.A., Ugwu E.T., Mohammed I.Y. (2019). Pattern of obesity among patients with type 2 diabetes at a tertiary healthcare center in northern Nigeria. Diabetes Metab Syndr Obes.

[39] Golmohammadi M., Zarrin R., Ayremlou P. (2020). Adaptation and validity assessment of a diet quality index for patients with type 2 diabetes. J Diabetes Metab Disord.

[40] Cheung L.T.F., Chan R.S.M., Ko G.T.C., Lau E.S.H., Chow F.C.C., Kong A.P.S. (2018). Diet quality is inversely associated with obesity in Chinese adults with type 2 diabetes. Nutr J.

[41] Chetoui A., Kaoutar K., Boutahar K., El Kardoudi A., Elmoussaoui S., Chigr F. (2021). Prevalence of overweight/obesity and its associated factors among a sample of Moroccan type 2 diabetes patients. Afr Health Sci.

[42] Bizuayehu T., Menjetta T., Mohammed M. (2022). Obesity among type 2 diabetes mellitus at sidama region, southern Ethiopia. PLoS One.

[43] Endrias E.E., Feleke M.G. (2023). Magnitude of high body mass index and its associated factors among adult type 2 diabetes mellitus patients attending follow-up clinics at hospitals in Wolaita Zone, southern Ethiopia, 2021. Int J Afr Nurs Sci.

[44] Newson R.S., Divino V., Boye K.S., Chen J., DeKoven M., Vallarino C. (2024). Glycemic control and obesity among people with type 2 diabetes in Europe and Australia: a retrospective cohort analysis. Diabet Ther.

[45] Borgharkar S.S., Das S.S. (2019). Real-world evidence of glycemic control among patients with type 2 diabetes mellitus in India: the TIGHT study. BMJ Open Diabet Res Care.

[46] Yosef T., Nureye D., Tekalign E. (2021). Poor glycemic control and its contributing factors among type 2 diabetes patients at adama hospital medical college in east Ethiopia. Diabetes Metab Syndr Obes.

[47] Feleke B.E., Feleke T.E., Kassahun M.B., Adane W.G., Fentahun N., Girma A. (2021). Glycemic control of diabetes mellitus patients in referral hospitals of amhara region, Ethiopia: a cross-sectional study. Biomed Res Int.

[48] Tekalign A.M., Lambero H., Motuma A. (2025). Determinants of poor glycaemic control in type 2 diabetes in Dire Dawa city, eastern Ethiopia: a case–control study. Int Health.

[49] Mengstie M.A., Abebe E.C., Dejenie T.A., Seid M.A., Teshome A.A. (2024). Frequency and correlates of poor glycemic control in patients with type 2 diabetes at jimma medical centre, Ethiopia: a cross-sectional study. Pan Afr Med J.

[50] Wondmkun Y.T. (2020). Obesity, insulin resistance, and type 2 diabetes: associations and therapeutic implications. Diabetes Metab Syndr Obes.

[51] Boden G. (2011). 45Obesity, insulin resistance and free fatty acids. Curr Opin Endocrinol Diabetes Obes.

[52] Bays H.E. (2023). Why does type 2 diabetes mellitus impair weight reduction in patients with obesity? A review. Obes Pillars.

